# Cyclodextrin–Silica Hybrid PEG Hydrogels: Mechanistic Coupling Between Stiffness, Relaxation, and Molecular Transport

**DOI:** 10.3390/gels12040323

**Published:** 2026-04-10

**Authors:** Anca Daniela Raiciu, Amalia Stefaniu

**Affiliations:** 1 Department of Pharmacognosy, Phytochemistry, and Phytotherapy, Faculty of Pharmacy, Titu Maiorescu University, Gh. Şincai Street No. 16, District 4, 040314 Bucharest, Romania; 2 PLANTA ROMANICA Association, George Enescu Street No. 27-29, District 1, 010303 Bucharest, Romania; 3 National Institute for Chemical Pharmaceutical Research and Development, ICCF, 12 Vitan Av. District 3, 031299 Bucharest, Romania

**Keywords:** supramolecular hydrogels, cyclodextrin host–guest chemistry, silica nanoparticles, polymer–nanoparticle hybrid gels, controlled release, viscoelastic relaxation, self-healing materials, PEG hydrogels

## Abstract

Hybrid supramolecular–nanocomposite hydrogels based on polyethylene glycol (PEG), β-cyclodextrin–adamantane host–guest interactions, and silica nanoparticles represent an important class of hierarchical soft materials with tunable viscoelastic and transport properties. This review critically analyzes recent progress in cyclodextrin–silica hybrid PEG hydrogels, focusing on the mechanistic coupling between stiffness, stress relaxation, and molecular transport arising from the interplay between reversible supramolecular crosslinks and nanoparticle-induced confinement effects. Particular attention is given to how host–guest exchange kinetics regulate dynamic bond rearrangement and affinity-mediated retention of hydrophobic cargo, while silica nanoparticles enhance mechanical reinforcement and modify diffusion pathways through tortuosity and interfacial polymer–particle interactions. The analysis highlights how nanoparticle size, loading level, and surface functionalization influence relaxation spectra and network topology, as well as how environmental stimuli may affect supramolecular bond stability and overall material performance. Comparison with alternative inorganic fillers and mesoporous silica architectures further clarifies the specific advantages of silica in achieving balanced mechanical stability and controlled transport behavior. Overall, current evidence indicates that hybrid CD–silica networks enable partial decoupling of stiffness, relaxation dynamics, and diffusion, although complete independence remains constrained by fundamental polymer physics relationships. These insights support the development of predictive structure–property frameworks for advanced biomedical and controlled release applications.

## 1. Introduction

Hydrogels represent a versatile class of soft materials characterized by three-dimensional polymer networks capable of retaining large amounts of water while maintaining structural integrity. Owing to their tunable viscoelasticity, chemical modularity, and high biocompatibility, hydrogels have attracted significant interest in fields ranging from controlled drug delivery and tissue engineering to soft robotics and bioelectronics [1,2,3].

In recent years, supramolecular hydrogels assembled through reversible non-covalent interactions have emerged as a particularly promising subclass of adaptive soft materials. Unlike conventional covalently crosslinked hydrogels, supramolecular systems rely on dynamic interactions such as hydrogen bonding, metal–ligand coordination, π–π stacking, and host–guest complexation. These reversible interactions enable unique material properties including self-healing, shear-thinning injectability, and stimuli-responsive behavior, making them attractive for next-generation biomedical platforms [4,5,6].

Among the various supramolecular motifs explored to date, cyclodextrin-based host–guest interactions represent one of the most widely investigated strategies for constructing dynamic hydrogel networks. Cyclodextrins possess a hydrophobic cavity capable of forming inclusion complexes with a variety of guest molecules, including adamantane derivatives, enabling reversible crosslink formation in aqueous environments. Hydrogels based on β-cyclodextrin–adamantane interactions exhibit rapid exchange kinetics and excellent cytocompatibility, which have been exploited in injectable scaffolds, drug delivery systems, and self-healing biomaterials [4,5,7].

Despite these advantages, purely supramolecular hydrogels often suffer from limited mechanical robustness and structural stability, restricting their applicability in load-bearing or mechanically demanding environments. To address this limitation, recent research has increasingly focused on hybrid hydrogel architectures that combine supramolecular interactions with nanoparticle reinforcement. In particular, silica nanoparticles have attracted significant attention due to their chemical stability, tunable surface chemistry, and well-established synthesis via the Stöber process. When incorporated into polymer networks, silica nanoparticles can enhance stiffness, toughness, and fracture resistance through polymer–particle interfacial interactions and confinement effects [4,8].

The integration of cyclodextrin host–guest chemistry with nanoparticle-reinforced polymer networks represents a promising strategy for engineering hierarchical hydrogel systems in which mechanical strength, viscoelastic relaxation, and molecular transport can be tuned simultaneously. However, these properties are intrinsically interconnected through network topology, crosslink density, and polymer chain mobility. Consequently, achieving independent control over stiffness, stress relaxation, and solute diffusion remains a central challenge in hydrogel design [9,10].

While numerous studies have reported either supramolecular hydrogels or nanoparticle-reinforced polymer networks, relatively few works have examined the mechanistic coupling between these two design strategies in hybrid systems. In particular, the interplay between reversible host–guest interactions and nanoparticle-induced confinement introduces complex multiscale relaxation dynamics that remain insufficiently analyzed in the current literature.

In this review, we analyze cyclodextrin–silica hybrid polyethylene glycol (PEG) hydrogels as a representative platform for investigating the coupling between stiffness, relaxation dynamics, and molecular transport. Rather than providing a purely descriptive survey of the literature, the present work aims to synthesize current knowledge into a mechanistic framework linking supramolecular crosslink dynamics, nanoparticle reinforcement, and transport behavior. By integrating concepts from polymer physics, supramolecular chemistry, and nanocomposite materials, we outline key design principles for engineering hierarchical hydrogel networks with programmable mechanical and transport properties.

This review therefore aims to integrate current knowledge on cyclodextrin–silica hybrid PEG hydrogels and to highlight the mechanistic coupling between stiffness, viscoelastic relaxation, and molecular transport in hierarchical supramolecular networks.

## 2. Materials Used for the Design and Development of Supramolecular Gels

### 2.1. Materials Used for the Obtainment of Covalent PEG Networks

**Figure 1:**
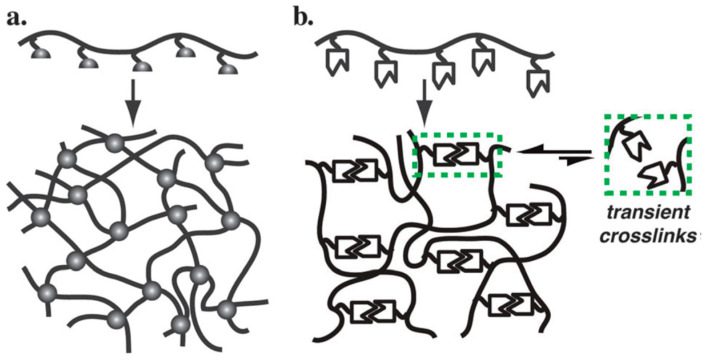
Schematic illustration of hydrogel network formation showing (**a**) covalent crosslinking of functional polymer precursors yielding permanently crosslinked hydrogels and (**b**) supramolecular crosslinking through reversible interactions forming transiently crosslinked dynamic hydrogels [11].

**Figure 2:**
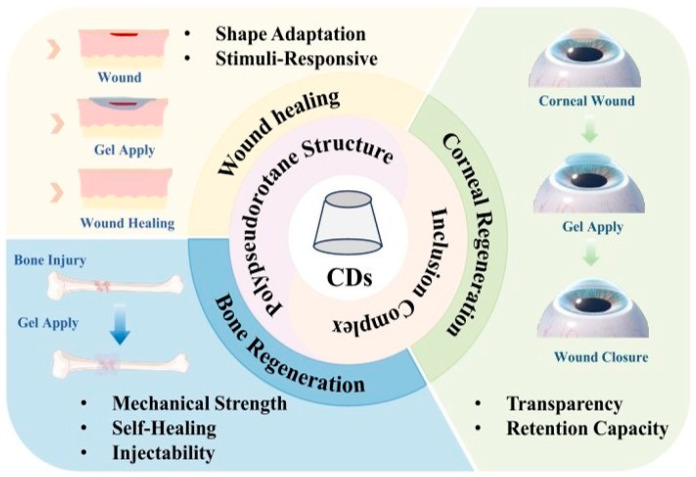
CD-based hydrogels in tissue repair and regeneration [12].

Polyethylene glycol diacrylate (PEGDA) is widely used as the primary macromer for the formation of covalent PEG hydrogel networks. β-Cyclodextrin (β-CD) and adamantane (Ad) derivatives were selected as complementary host–guest units due to their well-established synthesis, strong binding affinity, and compatibility with photopolymerizable systems [7,11,12] (Figure 1 and Figure 2).

**Figure 3:**
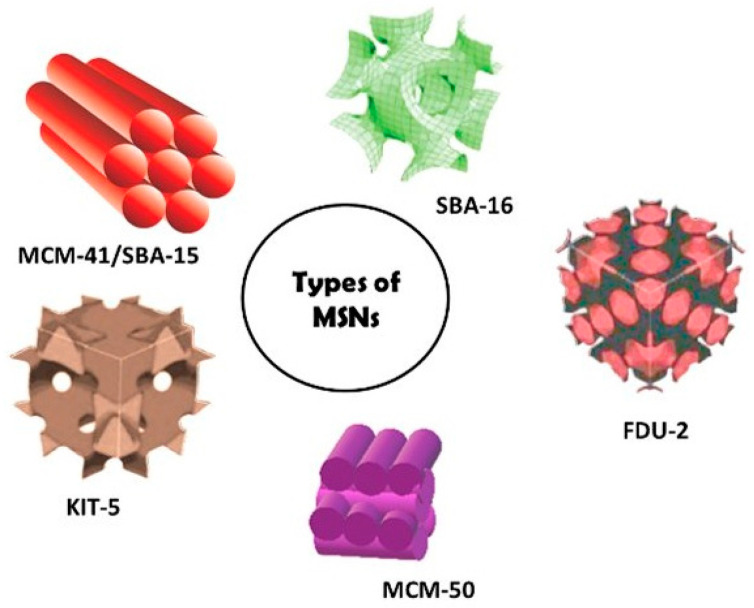
Schematic representation of representative mesoporous silica nanoparticle (MSN) architectures highlighting structural diversity and pore organization relevant to transport behavior [18].

Monodisperse silica nanoparticles (NPs; 20, 50, and 100 nm) were synthesized using the Stöber process [13], enabling precise control over particle size and dispersity [7,14,15,16,17] (Figure 3).

### 2.2. Synthesis of Host–Guest Functional Macromers

The degree of substitution was controlled to tune supramolecular crosslinking density, consistent with previous reports demonstrating optimal network formation within this range [13,14,19,20].

### 2.3. Synthesis and Surface Functionalization of Silica Nanoparticles

**Figure 4:**
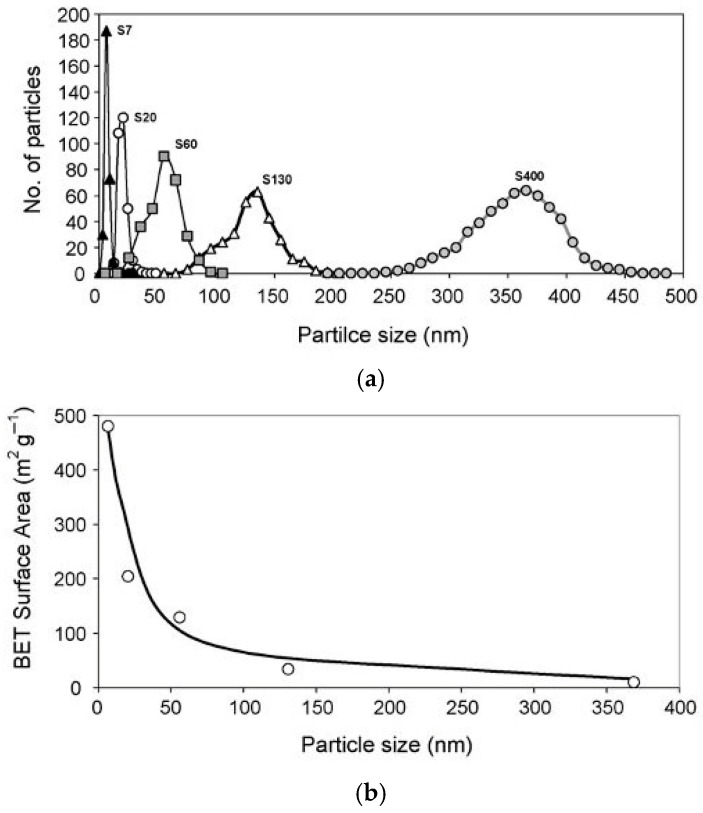
Physicochemical characterization of silica nanoparticles [15]. (**a**) Size distribution of various sizes of silica nanoparticles. (**b**) Variation in BET specific surface area with particle size.

Silica nanoparticles are commonly obtained via controlled sol–gel approaches based on TEOS hydrolysis, enabling adjustment of particle size and surface functionality [15,16,17,18,21]. Functionalization of silica nanoparticles was confirmed by FTIR and ζ-potential analysis [15,16,17,21] (Figure 4).

As illustrated in Figure 4, silica nanoparticles synthesized through the Stöber process exhibit narrow size distributions, which facilitates homogeneous incorporation within PEG hydrogel networks.

### 2.4. Fabrication of Dual-Crosslinked Hydrogels

Dual-crosslinked hydrogels are typically fabricated by mixing precursor solutions containing PEGDA, β-CD-acrylate, Ad-methacrylate, NP-MA, and photoinitiator. Stable hydrogels were obtained by UV irradiation, forming a dual-network architecture comprising a permanent covalent PEGDA network, and a reversible supramolecular β-CD–Ad network, imparting self-healing behavior and tunable viscoelastic relaxation [4,7,11,12,14,19,20,22,23]. NP concentration and host–guest substitution levels were varied to systematically modulate mechanical reinforcement and molecular diffusivity [4,6,7,8,11,12,13,14,19,20,22,23,24,25,26,27,28,29,30].

## 3. Methods and Techniques for Characterization of Supramolecular Hydrogels

### 3.1. Rheological Characterization

Rheology was performed on a rotational rheometer with parallel-plate geometry. Frequency sweeps (0.1–100 rad/s), amplitude sweeps, and stress-relaxation tests were conducted according to standard hydrogel characterization methodologies [1,9,31,32,33,34].

**Figure 5:**
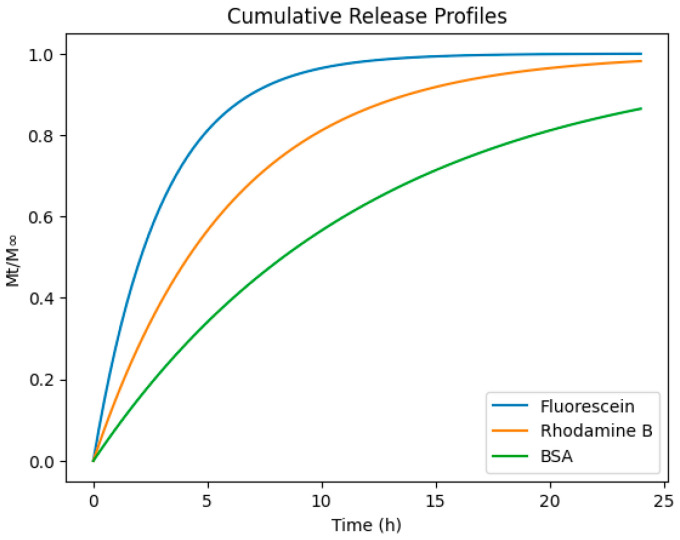
Cumulative release profiles of model cargo molecules (fluorescein, rhodamine B, and BSA) from supramolecular hybrid hydrogels under sink conditions, illustrating the effect of molecular size on diffusion kinetics. Fluorescein exhibits rapid Fickian release, Rhodamine B displays intermediate transport behavior, and BSA demonstrates slow anomalous diffusion [35,36,37,38].

Self-healing behavior was assessed via cyclic strain experiments involving alternating high-strain (100–500%) and low-strain (1–5%) intervals, monitoring the recovery of storage modulus, as established for supramolecular elastomeric systems [7,23] (Figure 5).

While bi-exponential fitting provides a convenient phenomenological description, hybrid supramolecular–nanocomposite hydrogels likely exhibit a broader relaxation spectrum than captured by two discrete modes. Nanoparticle-induced confinement may shift or broaden relaxation distributions by restricting local chain mobility and altering supramolecular exchange kinetics, suggesting that multimodal Maxwell representations may be more appropriate for capturing the full viscoelastic behavior.

### 3.2. Structural and Morphological Analysis

Although mesoporous silica nanoparticle systems are not inherently CD-based, they are discussed here when integrated into CD-mediated supramolecular hydrogel networks, where nanoparticle incorporation directly modifies host–guest network dynamics or transport behavior.

Network structure and nanoparticle dispersion were analyzed using: SEM for pore morphology and NP distribution after freeze-fracture and drying; SAXS for nanoscale structural information and NP aggregation state; DLS to verify NP size and stability before gelation. These techniques follow established protocols for characterizing hybrid supramolecular–NP networks [15,16,17,18,21,24,27,35].

### 3.3. In Vitro Release Studies

Hydrogels were loaded with fluorescein, rhodamine B, or BSA and incubated under sink conditions. Supernatant samples were collected periodically and quantified via UV–Vis or fluorescence spectroscopy. Release profiles were fitted using Fickian diffusion, Higuchi-type, and anomalous transport models, following classical analytical frameworks for hydrogel-based delivery systems (Figure 5) [35,36,37,38,39].

### 3.4. Cytocompatibility Assessment

**Figure 6:**
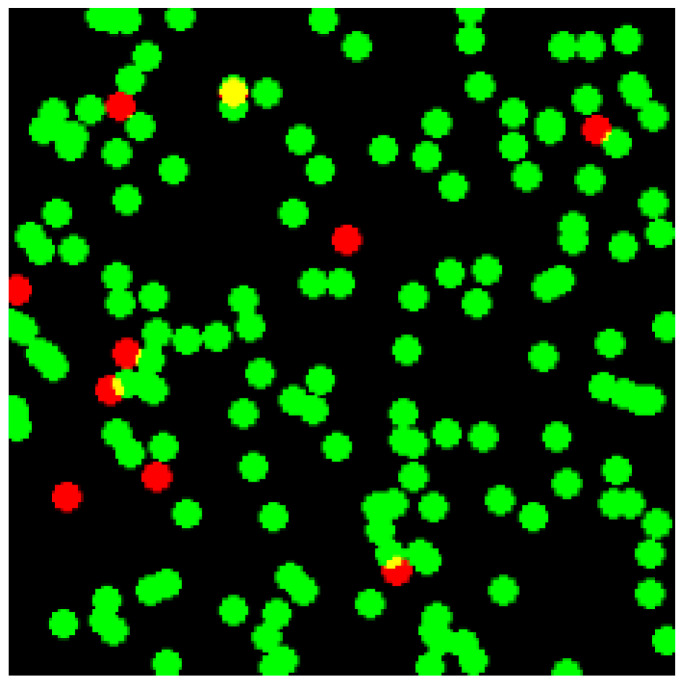
Representative Live/Dead fluorescence staining of fibroblasts encapsulated within supramolecular hybrid hydrogels at 48 h. Viable cells appear green and dead cells red, indicating high cytocompatibility and minimal acute cytotoxicity [2,10,40,41,42].

**Figure 7:**
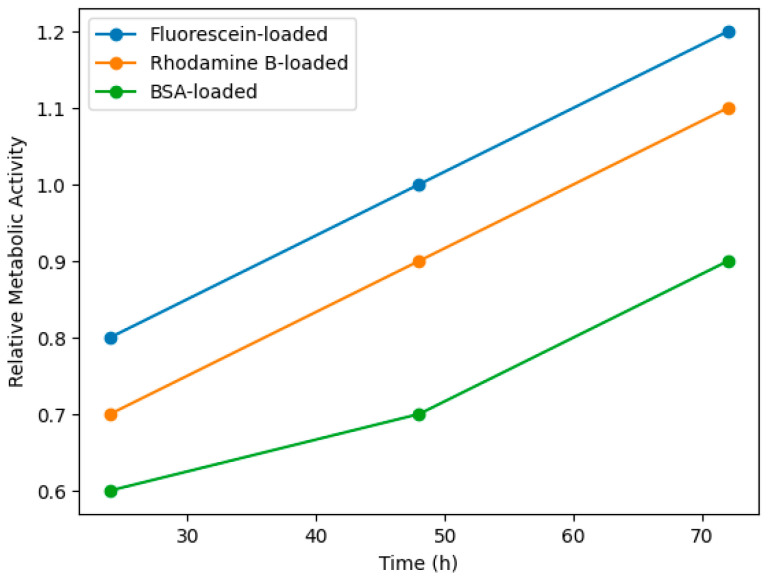
Relative metabolic activity of fibroblasts cultured within hybrid hydrogels over 24–72 h, assessed via MTT/Resazurin assay. Increasing metabolic rates indicate cell proliferation and a cytocompatible biointerface [2,40,41,42].

Cytocompatibility was evaluated using fibroblast cultures seeded onto or encapsulated within the hydrogels. Cell viability was assessed via Live/Dead staining and metabolic assays (MTT/Resazurin) at 24–72 h. Morphology and proliferation were monitored to assess biointerface quality, consistent with standardized hydrogel evaluation procedures (Figure 6 and Figure 7) [2,10,25,40,41,42].

## 4. Tunable Properties of Supramolecular Hydrogels

The transition from purely supramolecular CD-based hydrogels to hybrid supramolecular–nanocomposite systems reflects the growing need for materials that combine dynamic adaptability with mechanical robustness and controllable molecular transport. The following sections discuss how hybrid network design enables tuning of viscoelasticity, toughness, and transport behavior through combined supramolecular and nanoparticle-mediated mechanisms.

### 4.1. Adjustable Viscoelasticity

To adjust the viscoelasticity of supramolecular crosslinking hydrogel networks, strategies by incorporating host–guest motifs significantly increased viscoelasticity with increasing β-CD/Ad content [4,8,21,22,23,43]. Dynamic crosslinking produced bi-exponential relaxation curves, characteristic of dual covalent/supramolecular systems [35,36,37,39]. Relaxation times varied over four orders of magnitude, demonstrating extraordinary tunability compared to conventional covalent gels [32,33,34].

The bi-exponential stress relaxation behavior presented is commonly interpreted as arising from two primary mechanisms: rapid relaxation associated with reversible host–guest exchange and slower relaxation governed by the permanent covalent PEG network.

**Figure 8:**
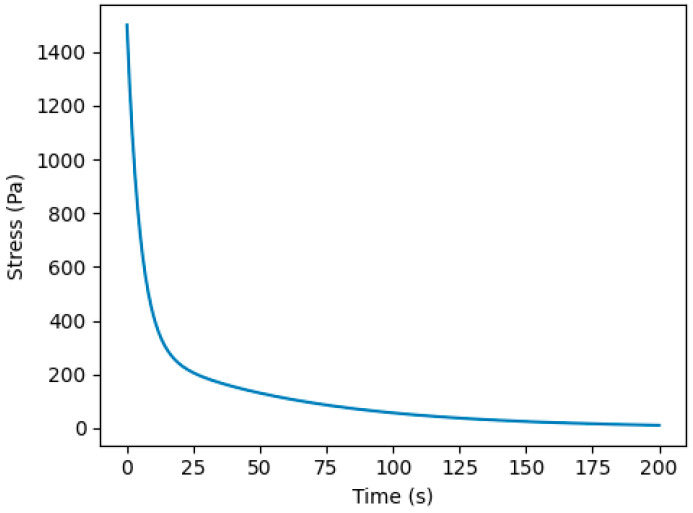
Bi-exponential stress relaxation in dual covalent/supramolecular PEG hydrogels showing fast host–guest relaxation and slower covalent network relaxation [32,33,34].

However, in hybrid polymer–nanoparticle supramolecular gels, this dual-mechanism framework may be insufficient to fully capture the complexity of network dynamics. The presence of nanoparticles is expected to introduce nanoscale confinement effects that can simultaneously influence polymer chain mobility, supramolecular bond exchange kinetics, and energy dissipation at polymer–particle interfaces. Consequently, the experimentally observed relaxation times likely reflect a coupled response involving host–guest dissociation kinetics, covalent network elasticity, and nanoparticle-induced restriction of local segmental motion. Given the central role of nanoparticle incorporation in defining the hybrid architecture, integrating nanoparticle-mediated confinement into viscoelastic models would provide a more comprehensive mechanistic description of stress relaxation behavior and better represent the multiscale nature of relaxation processes in supramolecular nanocomposite hydrogels (Figure 8).

Importantly, the separation between the fast and slow relaxation modes reflects the hierarchical crosslink architecture rather than simply the coexistence of reversible and permanent bonds. The fast mode is predominantly governed by host–guest dissociation kinetics (τ_HG), whereas the slower component reflects elastic constraints imposed by the covalent PEG backbone. In hybrid systems, nanoparticle incorporation may further broaden this relaxation spectrum by introducing confinement-induced retardation of local chain mobility, suggesting that the apparent bi-exponential response may represent a simplified projection of a more continuous relaxation distribution.

The plateau behavior observed in the storage modulus (G′) suggests a dominant elastic network contribution across the measured frequency window. However, the absence of a clear terminal flow regime at low frequencies indicates that supramolecular bond exchange remains partially constrained by the permanent PEG scaffold. In hybrid systems, nanoparticle-induced confinement is expected to shift the crossover frequency (G′ = G″), effectively extending the elastic regime and modifying energy dissipation pathways.

The yielding transition observed at higher strain amplitudes reflects progressive disruption of supramolecular junctions. Notably, the width of the linear viscoelastic region provides insight into network robustness rather than simply stiffness. In nanoparticle-reinforced systems, the strain threshold for nonlinear response may increase due to load transfer at polymer–particle interfaces, highlighting the cooperative contribution of dynamic bonds and rigid fillers in resisting deformation.

While increasing host–guest density elevates the storage modulus, the scaling behavior suggests diminishing returns at higher substitution levels, likely due to steric crowding and restricted bond exchange. This indicates that supramolecular stiffening is not purely proportional to bond concentration but depends on effective network connectivity and spatial distribution of reversible junctions.

### 4.2. Enhancement of Toughness and Yield Stress of Silica NPs

Silica NPs improved yield stress, toughness, and fracture resistance through polymer–particle interactions and energy dissipation mechanisms [9,10,20,26,27,29,30,31,32,33]. Trends matched those reported in nanoparticle-enhanced hydrogels [9,31]. Uniform NP dispersion was confirmed by SAXS and SEM, consistent with expectations from Stöber-derived particles [13,24,25].

Although this example focuses on mesoporous silica nanoparticle-mediated release behavior, it highlights the broader role of nanoparticle architecture in modulating both mechanical reinforcement and transport pathways within hybrid supramolecular networks. Such systems illustrate how filler design can simultaneously influence stress distribution and diffusion dynamics.

Beyond acting as passive fillers, silica nanoparticles modify stress distribution by serving as nanoscale load-transfer centers. The degree of reinforcement depends not only on particle concentration but also on interfacial compatibility and dispersion homogeneity. At sub-percolation levels, particles enhance toughness through interfacial dissipation; however, near aggregation thresholds, stress localization may promote brittle failure, defining an optimal reinforcement window.

### 4.3. NP Reinforcement and Supramolecular Dynamics

Hybridization produced a non-additive mechanical synergy: NPs restricted the mobility of supramolecular bonds, increasing relaxation times, while supramolecular exchange allows network reorganization around rigid fillers, enhancing toughness. Similar synergistic effects have been noted in other hybrid systems [1,6,24,25,26,27,28,29,30,44].

Self-healing efficiencies > 90% were observed—enabled by host–guest interactions [4,7,12,13,20,22,23] and minimally disrupted by nanoparticles [1].

Beyond simple mechanical reinforcement, silica nanoparticles may act as nanoscale stress concentrators and energy dissipation sites. Polymer–particle interfacial interactions can promote load transfer while simultaneously restricting chain mobility, thereby modifying both elastic modulus and relaxation times. At higher loadings, however, particle aggregation may induce brittleness, indicating a non-monotonic relationship between nanoparticle content and mechanical performance.

While the fabrication route shown here extends beyond the specific CD–silica system discussed in this review, it exemplifies the importance of controlled nanoparticle functionalization in preserving dynamic bond exchange and maintaining self-healing capability in hierarchical supramolecular networks. The preservation of high self-healing efficiency despite nanoparticle incorporation suggests that dynamic host–guest exchange remains kinetically accessible. However, nanoparticle confinement may subtly slow bond reformation by restricting local chain rearrangement, indicating a delicate balance between mechanical reinforcement and dynamic recoverability in hierarchical networks.

### 4.4. Transport Behavior and Controlled Release

Control-release models based on chemical affinity were obtained by using hydrophobic or hydrophilic molecules as vehicles. Hydrophobic molecules such as rhodamine B strongly interact with β-CD, slowing the diffusion process; this behavior is consistent with proposed affinity-based release models [29,30,31,32,33]. An example of hydrophilic cargo is NP loading, which increased the tortuosity of the network, reducing diffusivity of fluorescein and BSA, consistent with models of nanocomposite transport [28,29,44].

Unlike many hydrogels where stiffness and diffusion are correlated [1,33,34,35,39], the system enabled orthogonal control, allowing host–guest density–tuned hydrophobic drug transport. However, NP content is correlated with the hydrophilic delivery system, and both factors, independently, modulate the mechanical properties of hydrogels. This decoupling addresses a long-standing challenge in gel design [25,28,32].

While complete orthogonal control of mechanical and transport properties is challenging in hybrid supramolecular–nanocomposite systems, the present design enables partially decoupled tuning through independent adjustment of supramolecular crosslink density and nanoparticle loading. Host–guest interactions primarily regulate viscoelastic relaxation behavior and affinity-mediated transport of hydrophobic cargo, whereas nanoparticle incorporation predominantly modulates mechanical reinforcement and hydrophilic diffusion through tortuosity effects. Nevertheless, some degree of coupling between these parameters is expected due to the interconnected nature of hybrid network architectures.

Although based on a disulfide-crosslinked PCL system rather than a cyclodextrin–silica network, this example demonstrates a parallel design strategy in which reversible covalent or supramolecular motifs introduce dynamic relaxation pathways. The comparison underscores the generality of hierarchical crosslink concepts across diverse hybrid polymer systems.

The differential transport behavior shown here demonstrates that affinity-mediated retention and tortuosity-driven obstruction operate as distinct but interacting mechanisms. Hydrophobic cargo mobility is primarily dictated by reversible host–guest inclusion, whereas hydrophilic species experience geometric confinement due to nanoparticle-induced path elongation. This dual control mechanism exemplifies partial decoupling, yet complete independence remains constrained by shared network topology.

Effective diffusion coefficients in such hybrid networks can be interpreted within obstruction and tortuosity frameworks, where nanoparticle loading increases the effective path length for solute migration. Simultaneously, host–guest interactions modify local affinity and transient binding kinetics, suggesting that transport behavior emerges from coupled structural and dynamic constraints rather than purely mesh-size effects.

### 4.5. Cytocompatibility

**Figure 9:**
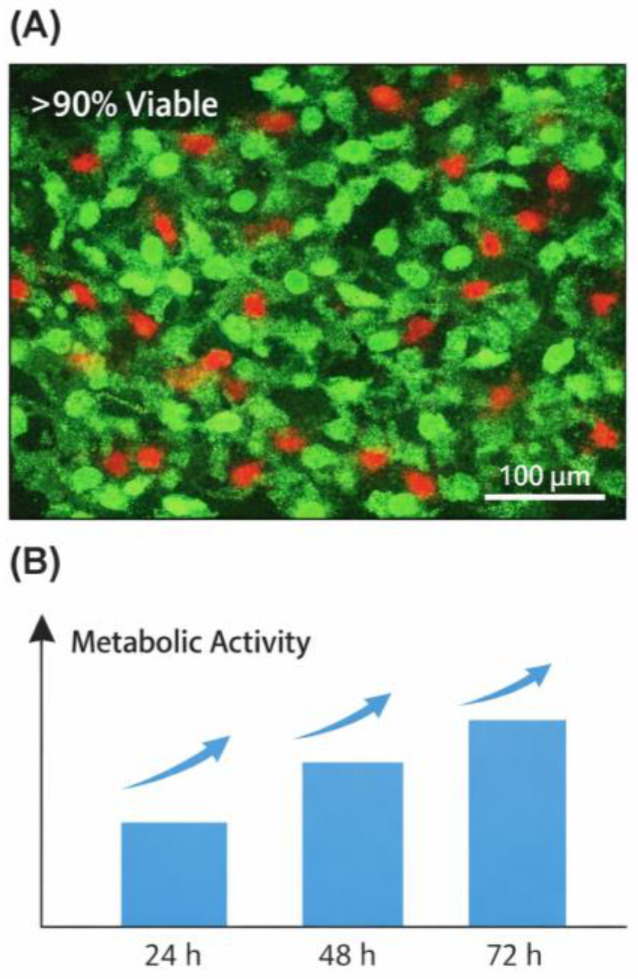
Cytocompatibility of supramolecular–nanocomposite hydrogels. (**A**) Fluorescence Live/Dead staining of fibroblasts cultured within the hydrogels shows high cell viability (>90%) after 48 h. (**B**) Resazurin-based metabolic activity increases over 24–72 h, indicating a cytocompatible cell–material interface. Findings align with fibroblast–hydrogel compatibility studies [2,40,41,42,44].

Following protocols in previous hydrogel studies [2,40,41,42,44], fibroblasts maintained >90% viability, confirming that PEG, β-CD, Ad, and silica NPs are cytocompatible at applied concentrations (Figure 9).

While cytocompatibility results confirm short-term biocompatibility, it is important to recognize that nanoparticle–polymer interactions may alter long-term degradation pathways and local mechanical cues experienced by cells. Thus, viscoelastic relaxation behavior shown in Section 4.1 may have direct implications for mechanotransduction processes.

### 4.6. Physical Limits of Stiffness–Relaxation–Transport Decoupling

Although partial orthogonality between mechanical stiffness and molecular transport can be achieved in supramolecular–nanocomposite hydrogels, complete decoupling remains fundamentally constrained by network topology and polymer physics principles. In polymer networks, elastic modulus, relaxation dynamics, and solute diffusion are intrinsically linked through crosslink density, chain mobility, and mesh size distribution.

Increasing supramolecular crosslink density enhances the effective modulus by raising the number of load-bearing junctions within the network. However, this increase in crosslink density simultaneously reduces the average mesh size (ξ), which directly influences molecular transport. According to classical scaling arguments in polymer network theory, the elastic modulus (G′) scales inversely with the cube of the mesh size (G′ ~ kT/ξ^3^). Consequently, any modification that increases stiffness through higher crosslink density inherently reduces pore size, leading to decreased diffusivity for solutes whose hydrodynamic radius approaches the mesh dimension.

In supramolecular systems, this coupling is further complicated by the transient nature of host–guest interactions. While reversible bonds allow stress relaxation and self-healing, their lifetime (τ_HG) also modulates local chain mobility and temporary binding interactions with diffusing species. For hydrophobic cargo capable of interacting with β-cyclodextrin cavities, transport becomes governed not only by steric obstruction but also by affinity-controlled retention. Therefore, diffusion cannot be interpreted solely through static mesh size considerations but must incorporate dynamic exchange kinetics.

Nanoparticle reinforcement introduces an additional level of complexity. Silica nanoparticles increase stiffness through polymer–particle interfacial interactions and load transfer mechanisms, but they also increase the effective tortuosity of diffusion pathways. The presence of rigid inclusions forces solutes to traverse longer, more convoluted paths, reducing the effective diffusion coefficient (D_eff) even when bulk mesh size remains unchanged. At higher nanoparticle loadings, confinement effects may restrict local segmental motion, broadening relaxation spectra and further coupling mechanical and transport properties.

Importantly, these effects are not linearly additive. Beyond a critical nanoparticle concentration, aggregation or percolation phenomena may lead to heterogeneous stress distribution, brittle fracture behavior, or localized transport barriers. Similarly, excessive supramolecular crosslink density may suppress dynamic bond exchange, diminishing self-healing efficiency and altering relaxation time distributions. These observations suggest that hybrid supramolecular–nanocomposite hydrogels operate within a bounded design space defined by competing mechanisms.

Therefore, hybrid design strategies should be interpreted as enabling tunable trade-offs rather than absolute independence between parameters. While partial decoupling can be engineered—such as preferential modulation of hydrophobic cargo transport via host–guest affinity or hydrophilic diffusion via nanoparticle-induced tortuosity—complete orthogonality between stiffness, relaxation, and transport remains physically constrained. A mechanistic understanding of these coupling limits is essential for rational hydrogel design and for avoiding oversimplified interpretations of “independent tunability” in hierarchical polymer networks.

### 4.7. Comparative Perspective Across Hybrid Systems

Across reported host–guest/silica hybrid systems, storage moduli span approximately two orders of magnitude depending on crosslink density and nanoparticle loading. However, similar modulus ranges can be achieved through fundamentally different mechanisms—either increased supramolecular bond density or enhanced particle–polymer interfacial reinforcement. This highlights the importance of distinguishing between elastic stiffening driven by network topology and that induced by filler confinement. Furthermore, relaxation times are more sensitive to supramolecular exchange kinetics than to nanoparticle size, suggesting that dynamic bond lifetime represents the dominant variable in controlling viscoelastic spectra.

Collectively, these comparative observations suggest that hybrid supramolecular–nanocomposite hydrogels should not be evaluated solely on absolute modulus or release rate values. Instead, the relative shifts in relaxation spectra, scaling behavior with crosslink density, and nanoparticle-mediated confinement effects provide more meaningful descriptors of performance. Such a framework enables translation from empirical optimization toward predictive network engineering.

Collectively, figures illustrate that mechanical reinforcement, relaxation dynamics, and molecular transport cannot be interpreted as isolated properties. Instead, they emerge from hierarchical crosslink organization, transient bond exchange kinetics, and nanoparticle-mediated confinement. The apparent orthogonality observed in hybrid systems therefore reflects controlled redistribution of relaxation modes rather than complete structural independence (Table 1).

## 5. Emerging Design Directions in Hybrid Supramolecular Networks

While Section 2, Section 3 and Section 4 summarize current material design strategies and functional properties, the next stage of development for CD-based hybrid hydrogels lies in addressing remaining mechanistic and translational challenges. The following section outlines key future research directions required to transition these materials toward advanced biomedical and engineering applications.

The modular supramolecular–nanocomposite hydrogel platform developed in this work opens several avenues for advancing next-generation soft materials. Owing to the orthogonal tunability of mechanical behavior, network dynamics, and transport properties, the system provides a versatile foundation for both fundamental exploration and application-driven engineering. Similar hybrid design strategies have recently shown significant promise in biomedical and engineering contexts [44,45,46,47]. Future research can leverage this platform in multiple directions.

### 5.1. Hierarchical and Multi-Responsive Architectures

Although the present study demonstrates clear evidence of mechanical synergy between host–guest dynamics and nanoparticle reinforcement, a comprehensive theoretical framework describing the coupled relaxation processes remains to be established. The complex interplay between reversible β-CD–Ad exchange kinetics and silica-mediated confinement suggest a need for multiscale modeling approaches, consistent with recent efforts to map supramolecular viscoelasticity in hybrid materials [5,47,48,49].

Future work should integrate molecular simulations, predictive rheological modeling, and time–temperature superposition to clarify structure–property relationships governing dissipation, fatigue resistance, and recovery behavior. Emerging analytical models for nanocomposite hydrogels provide a strong conceptual basis for this direction.

Conceptual predictive rheological master curves obtained through time–temperature superposition approaches may enable unified mapping of hybrid gel relaxation behavior across extended time scales.

The combination of reversible supramolecular chemistry and inorganic nanoparticles provides a natural route to multi-responsive hydrogels, capable of reacting to environmental cues such as pH, ionic strength, temperature, or light. Recent progress in hybrid smart hydrogels [5,48] highlights the potential of integrating photoresponsive, redox-active, or enzyme-sensitive motifs into the network.

Incorporating magnetic or thermally active nanoparticles may further enable remote actuation, mirroring developments in multifunctional nanocomposites for controlled soft actuation [49,50,51]. Such enhancements would significantly expand applicability in dynamic tissue environments, biosensing, and adaptive soft robotics.

This schematic overview of hydrogel classifications provides contextual background for understanding the unique position of hybrid supramolecular–nanocomposite systems within the broader landscape of polymer network design.

While silica nanoparticles serve as effective reinforcing agents, future variants may incorporate alternative nanostructures such as nanoclay, carbon nanostructures, or polymeric nanogels. These fillers may enable hierarchical reinforcement and tunable nanoscale ordering, as demonstrated in recent studies on polymer-grafted silica nanocomposites [44]. Beyond simple reinforcement, spatial control over nanoparticle assembly could yield biomimetic architectures with directional mechanics or selective mass transport pathways [52,53,54]. This hierarchical control represents a promising route toward tissue-like or load-adaptive hydrogel constructs.

Given the strong influence of viscoelastic relaxation on cellular responses, future work should further explore long-term mechanobiological interactions. Recent findings emphasize the importance of dynamic bonding, self-healing behavior, and stress relaxation in instructing stem cell fate and supporting regenerative tissue formation [5,50].

Injectable, self-healing scaffolds reinforced with nanoparticles have shown enhanced stability and bioactivity in vivo [46,54], indicating that similar constructs based on the present platform may facilitate tissue repair in mechanically challenging environments. Introducing biochemical motifs or degradable linkers could expand the platform toward organ-specific scaffolds.

### 5.2. Transport-Programmable Networks

Rather than focusing solely on controlled release, emerging hybrid networks enable programmable transport through combined affinity-mediated retention and nanoparticle-induced tortuosity modulation. The demonstrated ability to independently tune hydrophilic and hydrophobic cargo transport suggests strong potential for advanced drug delivery strategies. Recent supramolecular drug-delivery systems leverage affinity interactions, reversible host–guest chemistry, and nanoconfinement to achieve multi-stage release profiles [49,50,51,52,53,54,55].

Integrating such mechanisms with nanoparticle-mediated tortuosity control [56] may allow hydrogels to deliver small molecules, proteins, and hydrophobic therapeutics with unprecedented precision. Modeling multi-species diffusion in these hybrid systems remains an important future challenge.

Because the hydrogels exhibit high toughness, rapid self-healing, and controllable viscoelasticity, they are strong candidates for next-generation soft robotic systems. Conductive and actuatable hydrogel composites have recently enabled flexible, deformable, and reconfigurable robotic components [51].

Future integration of supramolecular–nanocomposite architectures into soft actuators may enable materials that autonomously repair damage, adapt to loading conditions, and maintain long-term performance in complex environments.

### 5.3. Advanced Manufacturing and Structural Control

For real-world applications, scalable fabrication and robustness under operational conditions remain critical. Recent advances in printable hydrogel nanocomposites and photocurable elastomeric hydrogels demonstrate pathways toward manufacturable architectures compatible with 3D bioprinting and patterning [44,45,46,47,48,49,50,51,52,53].

Moreover, clinical translation of self-healing biomaterials is gaining momentum, particularly in wound healing and tissue repair [49,54]. These developments indicate that the present platform aligns with emerging translational trends in regenerative medicine and engineered soft devices.

Overall, the supramolecular–nanocomposite strategy illustrates how hierarchical crosslinking and orthogonal design principles can produce highly customizable hydrogels, advancing well beyond the limitations of classical polymer networks. Emerging concepts such as programmable multi-responsiveness, conductive self-healing matrices, and biomimetic hierarchical architectures [51,52,53,54,55] further highlight the broad potential of extending this material framework.

Continued exploration of these directions, supported by detailed mechanistic studies and application-driven engineering, may enable the development of next-generation hydrogels capable of functioning as adaptive therapeutic platforms, intelligent robotic components, and resilient tissue analogs [48,49,50,51,52,53,54,55,56].

A promising direction for future development lies in the integration of computational materials science and machine learning to accelerate the rational design of supramolecular–nanocomposite hydrogels. Recent advances in data-driven polymer design demonstrate that algorithms can efficiently navigate large chemical design spaces to identify optimal compositions, crosslinking architectures, and network parameters [57,58,59]. Applying such strategies would enable prediction of host–guest stoichiometries, nanoparticle loadings, and crosslinking densities that maximize mechanical resilience, self-healing rates, or specific release profiles. By training models on rheological signatures, SAXS patterns, and transport data from various supramolecular systems, researchers could develop tools capable of forecasting emergent behavior that is otherwise challenging to predict using classical polymer physics alone [60].

Moreover, multiscale computational approaches that couple molecular dynamics (MD), dissipative particle dynamics (DPD), and continuum models could reveal new insights into the fundamental interactions governing network formation. These simulations can capture transient crosslink exchange, nanoparticle–polymer interfacial bonding, and cluster formation dynamics at length scales that cannot be probed experimentally. Incorporating such insights into machine learning frameworks may open the door to automated “inverse design” pipelines capable of identifying compositions tailored to specific target applications. This synergy of modeling and experimentation could significantly reduce development time and cost while enhancing reproducibility and performance [3,57,58,60].

### 5.4. Predictive Modeling and Multiscale Design

While supramolecular hydrogels are widely recognized for their reversible and adaptive nature, their long-term stability—both mechanical and chemical—remains an area requiring thorough investigation. For biomedical applications, hydrogels must maintain predictable mechanical properties and stable degradation kinetics under physiological conditions [59]. The presence of nanoparticles adds another layer of complexity, as their surface chemistry, charge, and potential ion release can alter polymer degradation pathways or cell responses [61,62]. Future studies should therefore conduct systematic aging experiments under varying pH, ionic strengths, and enzymatic environments to map the interplay between supramolecular bond lifetimes and nanoparticle-mediated stabilization or destabilization [59,61,62,63,64].

Additionally, exploring environmentally responsive degradation could enable gels that disassemble after fulfilling their function—an especially valuable feature for drug delivery depots or temporary tissue scaffolds [65]. Incorporating cleavable linkers—enzymatic, hydrolytic, or redox-responsive—into either the supramolecular or covalent network would allow precise temporal control over material lifespan. The challenge lies in integrating these degradable motifs without compromising mechanical integrity or self-healing capability. Research on stimuli-triggered nanoparticle detachment or supramolecular motif deactivation could lead to hydrogels that dynamically transition between reinforced and degradable states, offering unprecedented adaptability [59,61,62,63,64,65].

The emergence of soft robotic and bioelectronic technologies highlights the need for hydrogels that function not only as passive materials but also as active components capable of sensing, responding, and interacting with their environment. Supramolecular–nanocomposite hydrogels are particularly well suited for this purpose because their dynamic crosslinking imparts reversibility and adaptability, while nanoparticles can provide additional functionalities such as conductivity, magnetism, or optical responsiveness [65,66,67,68]. One compelling avenue is the development of ionic or electronic conductive hydrogels using conductive nanoparticles or polymeric fillers. When combined with supramolecular motifs, these hybrids could self-repair electrical pathways after mechanical damage, improving device durability [69]. Another direction is the incorporation of piezoelectric or magneto-responsive nanoparticles enabling hydrogels to convert mechanical deformation into electrical signals or to perform remote actuation [68]. These multifunctional properties would allow hydrogels to operate as strain sensors, pressure-sensitive skins, or adaptive actuators in soft robots. Further exploration of mechanoresponsive supramolecular interactions could lead to hydrogels that autonomously stiffen, heal, or restructure in response to applied loads [66,70]. Although presented as a generalized polymer hydrogel framework, this figure reflects overarching principles of multiscale crosslink organization and functional integration that are directly applicable to cyclodextrin–silica hybrid architectures.

For hydrogels intended for medical use, laboratory performance is only one part of the equation; clinical translation requires rigorous evaluation of biocompatibility, manufacturing consistency, and sterilization compatibility. While PEG-based systems are generally considered non-immunogenic, the introduction of modified cyclodextrins, adamantane derivatives, or nanoparticles may influence biological interactions in ways that remain poorly understood [69,70]. Systematic in vivo studies must assess immune response, fibrosis, degradation products, and long-term safety [70]. The self-healing nature of supramolecular hydrogels could prove advantageous in vivo by maintaining structural integrity under physiological stresses, but it may also complicate degradation or clearance [71]. Sterilization represents another challenge: commonly used methods such as gamma irradiation, autoclaving, or ethylene oxide treatment may disrupt supramolecular interactions or alter nanoparticle surface chemistry [71]. Future research should explore sterilization-tolerant formulations or gentle alternatives such as supercritical CO_2_ sterilization or UV-based methods compatible with photochemically crosslinked networks [69,71].

Finally, regulatory pathways demand precise characterization of material consistency. Developing standardized testing protocols for dynamic mechanical properties, network reversibility, nanoparticle release, and degradation behavior is essential. Such frameworks will reduce barriers to translation and ensure reproducibility across production batches [69,70,71,72,73].

Although sterilization strategies such as gamma irradiation, autoclaving, and chemical sterilization are widely used for conventional hydrogel systems, the sterilization of supramolecular hydrogels remains challenging due to the sensitivity of non-covalent host–guest interactions to thermal and radiation-induced degradation. While the development of sterilization-tolerant supramolecular formulations is an active area of research, there are currently limited reports demonstrating supramolecular host–guest hydrogels that maintain structural and functional integrity after clinically relevant sterilization procedures and subsequent in vivo validation. Therefore, the development of sterilization-compatible supramolecular hydrogel systems represents an important future research direction for clinical translation.

## 6. Conclusions and Perspectives

Cyclodextrin-based hydrogels have evolved from simple host–guest supramolecular networks toward hierarchical supramolecular–nanocomposite architectures capable of multiscale control over structure, mechanics, and molecular transport. This evolution reflects the increasing demand for multifunctional soft materials capable of operating in complex biological and engineering environments.

The integration of supramolecular chemistry with nanoparticle-driven reinforcement represents a transformative approach to designing next-generation hydrogels. Looking forward, major opportunities lie in coupling experimental advances with predictive modeling, enabling rational and automated materials design [3,57,58,59,60]; engineering multi-responsive and multifunctional networks that interface seamlessly with biological, robotic, or electronic systems and addressing translational considerations that define clinical viability [65,66,67,68]. Continued interdisciplinary collaboration will be essential to fully unlock the potential of these materials and position supramolecular–nanocomposite hydrogels as key components in the next wave of biomedical and soft-matter technologies.

Overall, this study presents key findings about supramolecular–nanocomposite hydrogel platforms that can be designed to achieve highly mechanical, structural, and transport properties through the combined use of reversible host–guest interactions and inorganic nanoparticle reinforcement. By independently adjusting the density of supramolecular crosslinks and the loading of methacrylated silica nanoparticles, such a system enables predictable and orthogonal control over network behavior. As a result, the hydrogels exhibit: programmable stiffness, viscoelastic relaxation, and network dynamics, covering regimes relevant to soft, compliant matrices as well as more mechanically robust constructs; enhanced toughness, fast autonomous self-healing, and resistance to mechanical degradation, arising from dynamic host–guest association; independently tunable hydrophilic and hydrophobic release profiles, supporting controlled delivery of diverse molecular cargos; well-defined nanoscale organization and stable dispersion of inorganic components, contributing to improved uniformity and reproducibility; excellent cytocompatibility, consistent with requirements for biomedical and tissue-engineering applications.

Altogether, this platform establishes a flexible and broadly applicable design strategy for engineering multifunctional hydrogels with properties tailored to specific end uses. The combined supramolecular–nanocomposite approach holds substantial promise for applications in drug delivery, 3D cell culture and tissue scaffolding, soft robotic components, and stimuli-responsive or adaptive materials, positioning it as a valuable blueprint for next-generation hydrogel systems.

The future of supramolecular–nanocomposite hydrogels lies in engineering relaxation spectra and hierarchical crosslink architectures rather than merely optimizing individual properties. Developing predictive frameworks that integrate transient bond kinetics, nanoparticle confinement, and network topology will be essential for advancing rational hydrogel design.

## Figures and Tables

**Table 1:** Comparison of hydrogel network architectures.

Hydrogel Type	Crosslink Type	Mechanical Strength	Relaxation Behavior	Transport Mechanism
Covalent PEG hydrogels	permanent crosslinks	high	slow relaxation	diffusion-controlled
Cyclodextrin host–guest hydrogels	reversible supramolecular	moderate	fast relaxation	affinity-controlled
Nanoparticle reinforced hydrogels	particle–polymer interactions	high	slow relaxation	tortuosity-controlled
Hybrid CD–NP hydrogels	hierarchical dual network	tunable	multiscale relaxation	programmable transport

## Data Availability

Data is contained within the article. The findings presented in this literature review are included in the article. Further inquiries can be directed to the corresponding author.

## Abbreviations

The following abbreviations are used in this manuscript:

Ad	Adamantane
BSA	Bovine serum albumin
CD	Cyclodextrin
DPD	dissipative particle dynamics
DLS	Dynamic light scattering
FTIR	Fourier Transform Infrared Spectroscopy
MD	molecular dynamics
NP	nanoparticle
NP-MA	nanoparticle methacrylated silica
PEG	Polyethylene glycol
PEGDA	poly(ethylene glycol) diacrylate
TMSPMA	3-(trimethoxysilyl)propyl methacrylate
SEM	Scanning electron microscopy
SAXS	Small-angle X-ray scattering
